# Correction: Locality and diel cycling of viral production revealed by a 24 h time course cross-omics analysis in a coastal region of Japan

**DOI:** 10.1038/s41396-018-0247-1

**Published:** 2018-08-01

**Authors:** Takashi Yoshida, Yosuke Nishimura, Hiroyasu Watai, Nana Haruki, Daichi Morimoto, Hiroto Kaneko, Takashi Honda, Keigo Yamamoto, Pascal Hingamp, Yoshihiko Sako, Susumu Goto, Hiroyuki Ogata

**Affiliations:** 1 Graduate School of Agriculture, Kyoto University, Kitashirakawa-Oiwake, Sakyo-ku, Kyoto, 606-8502 Japan; 2 School of Life Science and Technology, Tokyo Institute of Technology, Ookayama, Meguro-ku, Tokyo, 152-8550 Japan; 3 Institute for Chemical Research, Kyoto University, Uji, Kyoto, 611-0011 Japan; 4 Research Institute of Environment, Agriculture and Fisheries, Osaka Prefecture, 442, Shakudo, Habikino, Osaka Japan; 5 Aix Marseille Université, Universitéde Toulon, CNRS, IRD, MIO UM 110, 13288 Marseille, France; 6 Database Center for Life Science, Joint-Support Center for Data Science Research, Research Organization of Information and Systems, Wakashiba, Kashiwa, Chiba 277-0871 Japan

Correction to: *The ISME Journal* (2018); 10.1038/s41396-018-0052-x; published online 30 January 2018

The original version of this Article contained an error in the main text citations and reference list. These errors have now been corrected in both the PDF and HTML versions of the Article.

